# Soluble klotho as an effective biomarker to characterize inflammatory states

**DOI:** 10.1080/07853890.2022.2077428

**Published:** 2022-05-23

**Authors:** Shou-En Wu, Wei-Liang Chen

**Affiliations:** a Department of Dermatology, Tri-Service General Hospital, School of Medicine, National Defense Medical Center, Taipei City, Taiwan; b Division of Family Medicine, Department of Family and Community Medicine, Tri-Service General Hospital, School of Medicine, National Defense Medical Center, Taipei City, Taiwan; c Division of Geriatric Medicine, Department of Family and Community Medicine, Tri-Service General Hospital, School of Medicine, National Defense Medical Center, Taipei City, Taiwan; d Department of Biochemistry, National Defense Medical Center, Taipei City, Taiwan

**Keywords:** Soluble Klotho, inflammation, uric acid, C-reactive protein, biomarker

## Abstract

**Background & Aims:**

Soluble α-Klotho (s-Klotho) is a circulating protein with pleiotropic effects that mainly induce protective effects. Our study investigates the associations between s-Klotho and several established inflammatory biomarkers, with the aim of examining whether s-Klotho levels are representative of inflammatory states.

**Methods:**

A total of 11,128 eligible participants from NHANES 2007–2016 were included in our study. Levels of four inflammatory biomarkers, uric acid (UA), C-reactive protein (CRP), white blood cell (WBC) count, and mean platelet volume (MPV), were examined for their relationship with s-Klotho levels. Sub-analyses sorted the total population by gender and into four quartiles. Linear regression models were used to evaluate the strengths of associations.

**Results:**

All four inflammatory biomarkers were significantly associated with s-Klotho levels. UA, CRP, and WBC count showed an inverse association, while MPV showed a direct one. Of the four markers, UA was most strongly correlated with s-Klotho levels (β coefficient: −28.89 in unadjusted model, *p*<.001), and this relationship was stronger in women than in men (β coefficient of UA in men: −22.01, *p*<.001; in women: −31.54, *p*<.001). In addition, all four biomarkers manifested stronger associations with s-Klotho in higher quartiles, and the highest absolute values of β coefficients appeared in Q4 vs. Q1.

**Conclusion:**

s-Klotho is significantly associated with well-recognized inflammatory biomarkers. A decrease in s-Klotho levels implies a general inflammatory status; therefore, s-Klotho serves as a potential biomarker that is inversely correlated with inflammatory conditions. Further applications in clinical practice will provide us with a better understanding of its role.

**Key messages**
- Soluble α-Klotho (s-Klotho) levels are significantly associated with the inflammatory markers uric acid, C-reactive protein, white blood cell count, and mean platelet volume.
- S-Klotho is involved in inflammatory processes and plays a protective role.
- S-Klotho may serve as an inverse indicator of inflammation.

## Introduction

The Klotho gene initially attracted scientists’ attention in 1997, when Makoto Kuro-o *et al.* identified that Klotho-deficient mice exhibited shortened lifespans and multiple aging-related phenotypes [1], suggesting that Klotho had anti-aging properties. The Klotho gene encodes a transmembrane α-Klotho protein, primarily found in renal tubules. The extracellular domain of the α-Klotho protein undergoes shedding and generates soluble α-Klotho (s-Klotho), which was reported to be a humoral factor with protective effects against systemic diseases, including chronic kidney disease [2], interstitial lung disease [3], and cardiovascular events [4]. In addition, studies have demonstrated that s-Klotho modulates oxidative stress [5,6], endothelial function [7], and cell senescence and apoptosis [8].

Emerging evidence illustrates a protective role of s-Klotho in various disorders, prompting researchers to investigate its underlying mechanisms. A study demonstrated that s-Klotho inhibits the insulin/insulin-like growth factor-1 signalling pathway, which promotes resistance to oxidative stress, thereby extending the lifespans of mice [9]. Other studies have depicted the anti-apoptotic and anti-senescence effects of s-Klotho in human umbilical vascular endothelial cells [8,10] and increased its antioxidant capacity in pulmonary epithelial cells [6], suggesting the modulation capability of s-Klotho in vasculature. s-Klotho also counteracts endothelial dysfunction by regulating NO availability [11], which exerts protective effects against cardiovascular events like atherosclerosis. Furthermore, s-Klotho manages calcium homeostasis by modulating the epithelial Ca^2+^ transient receptor potential vanilloid type 5 in nephrons, which contributes to the maintenance of normal renal function [12,13].

Because many studies have focussed on the benefits of s-Klotho in individual organs, there is less known about s-Klotho as a circulating factor and how it affects systemic health. This study explored the relationship between established inflammatory biomarkers and s-Klotho levels, aiming to determine whether s-Klotho modulates inflammation generally and its relationship with separate biomarkers.

## Materials and methods

### Study design and participants

The National Health and Nutrition Examination Survey (NHANES) 2007–2016 [14] database was used for this study. NHANES is a program hosted by the Centres for Disease Control and Prevention (CDC) and the National Centre for Health Statistics (NCHS) with the purpose of collecting health and nutritional data of adults and children in the United States (US). To ensure the samples are representative of the general US population, participants are randomly selected from across the country annually, and continuous release of datasets occurs in two-year cycles, except for a recent suspension due to the coronavirus disease-2019 pandemic. Before their data is entered into the survey, written informed consent is obtained from each participant. Our study protocol was approved by the NCHS Institutional Review Board. The two parts of the survey, in-house interviews and health examinations held at mobile examination centres, were performed by trained and qualified fellows.

**Figure 1.**
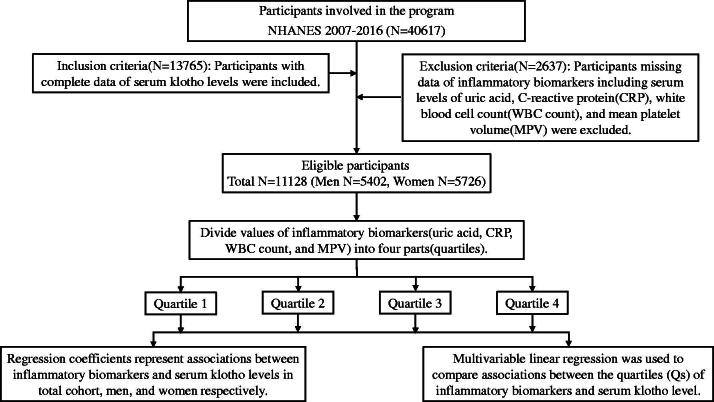
A flow diagram representing patient enrolment and study design of this study.

We collected information from NHANES 2007–2016 because the main biomarker of interest, s-Klotho, is available from these years. There were 40,617 participants between 2007 and 2016, and we initially included those with complete data about s-Klotho levels. Subsequently, we excluded those with missing data on inflammatory biomarkers, including serum levels of uric acid (UA) and C-reactive protein (CRP), white blood cell (WBC) count, and mean platelet volume (MPV), leaving 11,128 subjects eligible for further analyses. We performed analyses in the total cohort and in men and women separately to evaluate gender differences. In addition, we divided the values of inflammatory biomarkers into quartiles for detailed comparisons. A flowchart showing the design and structure of our study is presented in Figure 1.

### Measurement of serum biomarkers

All details about laboratory methodology and protocols are provided in the NHANES Laboratory/Medical Technologists Procedures Manual [15]. s-Klotho levels were examined using the IBL ELISA Kit (Immuno-Biological Laboratories, Gunma, Japan) according to the manufacturer’s instructions. Duplicate samples were conducted for each analysis, and the inter-assay coefficients of variation were 3.8% and 3.4%, respectively. The reference range for s-Klotho was 285.8–1638.6 pg/ml.

CRP concentration data included CRP values from 1999–2010 and high-sensitivity CRP from 2015–2018. The former values were determined using latex-enhanced nephelometry, and the latter was based on the highly sensitive near-infrared particle immunoassay rate methodology. WBC counts and MPVs are included in complete blood count tests. They were both analysed on the Beckman Coulter UniCel DxH 800 (Beckman Coulter, Miami, FL, USA). Albumin levels were measured using the DxC800 as a bichromatic digital endpoint method. High-density lipoprotein cholesterol levels were examined enzymatically with a Roche/Hitachi Modular P Chemistry Analyser (Roche Diagnostics GmbH, Mannheim, Germany) according to the manual.

### Covariates

Demographic information was acquired by trained examiners in household interviews. The formula for body mass index was kg/m^2^ (weight in kilograms/height in meters). The following categories for race were used: Mexican–American, other Hispanic, non-Hispanic White, non-Hispanic Black, and other races. A history of smoking was defined as having smoked at least 100 cigarettes in life. Histories of medical conditions were confirmed by asking the participants if they had ever been told by a doctor that they had one of the following conditions: coronary heart disease, angina, diabetes, and hypertension.

### Statistical analyses

SPSS (IBM SPSS Statistics for Windows, Version 22.0; IBM Corp., Armonk, NY, USA) was used for statistical analyses. Qualitative data were expressed in percentages (%), while quantitative variables were expressed as means and standard deviations. All *p* values<.05 were considered statistically significant. Linear regression analyses were utilized, and β coefficients were calculated to estimate the strengths of relationships. Three models were provided for each analysis to eliminate the effects of nuisance variables: Model 1 = unadjusted; Model 2 = adjusted for age, gender, body mass index, and race/ethnicity; Model 3 = Model 2 + adjusted for history of angina, coronary heart disease, diabetes mellitus, hypertension, and smoking status.

## Results

### Characteristics of participants

This study included 11,128 participants, including 5402 (48.5%) men and 5726 (51.5%) women. Demographic and laboratory profiles are shown in Table 1. The mean age of the entire cohort was 57.62 ± 10.89 years. The mean ± standard deviations of levels of inflammatory biomarkers of interest were the following: 7.11 ± 2.35 × 10^3^ cells/μl for WBC count, 862.29 ± 306.37 pg/ml for s-Klotho, 5.54 ± 1.43 mg/dl for UA, 0.46 ± 0.89 mg/dl for CRP, and 8.11 ± 0.95 fl for MPV.

**Table 1.** Characteristics of participants for total cohort, men, and women.

	Total cohort (*N* = 11,128)	Men (*N* = 5402, 48.5%)	Women (*N* = 5726, 51.5%)	*p* Value
Continuous variables				
Age (years)	57.62 ± 10.89	57.94 ± 10.87	57.40 ± 10.93	.009
BMI (kg/m^2^)	29.64 ± 6.68	29.16 ± 5.78	30.11 ± 7.39	<.001
Albumin (g/dl)	4.21 ± 0.31	4.27 ± 0.31	4.16 ± 0.31	<.001
HDL (mg/dl)	52.88 ± 16.36	47.89 ± 14.74	57.51 ± 16.49	<.001
White blood cell count (10^3^ cell/uL)	7.11 ± 2.35	7.17 ± 2.58	7.06 ± 2.11	.021
Klotho (pg/ml)	862.29 ± 306.37	838.63 ± 286.41	882.45 ± 321.46	<.001
Uric acid (mg/dl)	5.54 ± 1.43	6.07 ± 1.34	5.05 ± 1.34	<.001
C-reactive protein (mg/dl)	0.46 ± 0.89	0.41 ± 0.91	0.51 ± 0.87	<.001
Mean platelet volume (fL)	8.11 ± 0.95	8.07 ± 0.95	8.14 ± 0.95	<.001
Categorical variables				
Race				
Mexican American	1693 (15.2%)	817 (15.1%)	876 (15.3%)	.205
Other Hispanic	1186 (10.7%)	538 (10.0%)	648 (11.3%)	
Non-Hispanic White	5029 (45.2%)	2472 (45.8%)	2557 (44.7%)	
Non-Hispanic Black	2232 (20.1%)	1087 (20.1%)	1145 (20.0%)	
Other Race - Including Multi-Racial	988 (8.9%)	488 (9.0%)	500 (8.7%)	
History of coronary heart disease	548 (4.9%)	372 (6.9%)	176 (3.1%)	<.001
History of angina	346 (3.1%)	193 (3.6%)	153 (2.7%)	.016
History of diabetes	1724 (15.5%)	923 (17.1%)	801 (46.5%)	<.001
History of hypertension	2680 (24%)	1391 (27.4%)	1289 (24.7%)	.002
Smoking history	5461 (49.1%)	3180 (58.9%)	2281 (39.8%)	<.001

BMI: body mass index; HDL: high density lipoprotein.

Values in the continuous variables were expressed as mean and standard deviation.

Values in the categorical variables were expressed in number and percentage (%).

### Associations between serum inflammatory biomarker levels and s-Klotho levels

**Figure 2.**
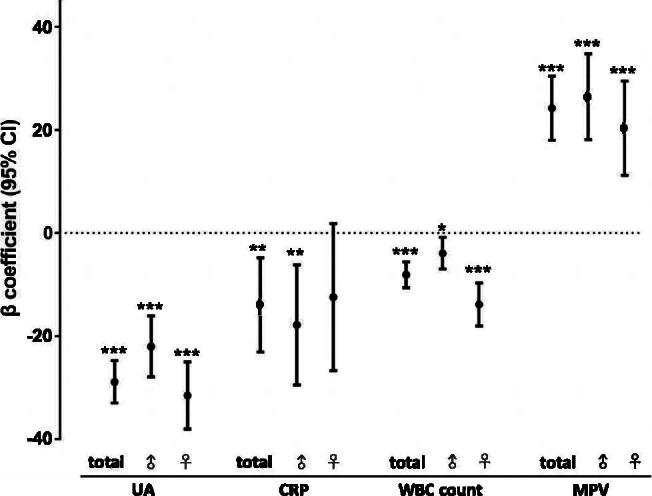
Forest plot of the β coefficients for the associations between inflammatory biomarkers and serum klotho levels in total cohort, men, and women. Linear regression was used to analyse the association between UA, CRP, WBC count, MPV and serum klotho levels. The β coefficient presents the degree of change in the serum klotho level for every 1-unit of change in the predictor variable. *p<.05, **p<.01, ***p<.001. UA: uric acid; CRP: C-reactive protein; WBC count: white blood cell count; MPV: mean platelet volume

As shown in Table 2 and Figure 2, UA, CRP, and WBC count were inversely associated with s-Klotho levels, while MPV was directly associated with s-Klotho levels. The β coefficient represents the degree of change in s-Klotho levels for one unit of change in the predictor variable. To compare the degree of relevance in the entire cohort, UA exhibited the largest absolute value of the β coefficient (β coefficient: −28.89 in the unadjusted model; 95% CI= −33.01, −24.77; *p*<.001). The remaining variables had the following β coefficients in descending order: MPV (β coefficient: 24.25 in the unadjusted model; 95% CI = 18.03, 30.46; *p* < .001); CRP (β coefficient: −13.93 in the unadjusted model; 95% CI= −23.10, −4.76; *p*=.003); and WBC count (β coefficient: −8.07 in the unadjusted model; 95% CI= −10.58, −5.56; *p*<.001). Further analyses showed gender differences for each biomarker. In women, UA and WBC count were more strongly correlated with s-Klotho levels ((UA: β coefficient of men: −22.01; 95% CI= −27.93, −16.07; *p*<.001; β coefficient of women: 31.54; 95% CI= −38.08, −25.01; *p*<.001) (WBC: β coefficient of men: −3.89; 95% CI= −6.92, −0.85; *p*=.012; β coefficient of women: −13.84; 95% CI= −18.03, −9.65; *p*<.001)). In men, CRP and MPV were more strongly correlated with s-Klotho levels ((CRP: β coefficient of men: −17.82; 95% CI= −29.48, −6.16; *p*=.003; β coefficient of women: −12.43; 95% CI= −26.70, 1.84; *p*=.088) (MPV: β coefficient of men: 26.49; 95% CI = 18.17, 34.82; *p*<.001; β coefficient of women: 20.38, 95% CI = 11.22, 29.55; *p*<.001)).

**Table 2.** Linear regression analyses for the associations between inflammatory markers and serum klotho levels in total cohort, men, and women.

		Model 1	Model 2	Model 3
Uric acid	Total			
	β coefficient (95% CI)	−28.89 (−33.01, −24.77)	−27.44 (−32.13, −22.76)	−26.57 (−31.25, −21.89)
	*P*-value	<.001	<.001	<.001
	Men			
	β coefficient (95% CI)	−22.01 (−27.93, −16.07)	−22.87 (−28.98, −16.76)	−21.29 (−27.41,−15.16)
	*P*-value	<.001	<.001	<.001
	Women			
	β coefficient (95% CI)	−31.54 (−38.08, −25.01)	−32.74 (−39.96, −25.52)	−31.77 (−38.99, −24.55)
	*P*-value	<.001	<.001	<.001
CRP	Total			
	β coefficient (95% CI)	−13.93 (−23.10, −4.76)	−14.64 (−23.92, −5.36)	−15.04 (−24.33, −5.76)
	*P*-value	.003	.002	.001
	Men			
	β coefficient (95% CI)	−17.82 (−29.48, −6.16)	−16.53 (−28.21, −4.85)	−16.96 (−28.63, −5.30)
	*P*-value	.003	.006	.004
	Women			
	β coefficient (95% CI)	−12.43 (−26.70,1.84)	−12.29 (−27.03,2.45)	−12.69 (−27.46,2.08)
	*P*-value	.088	.102	.092
WBC count	Total			
	β coefficient (95% CI)	−8.07 (−10.58, −5.56)	−8.22 (−10.76, −5.68)	−8.27 (−10.84, −5.70)
	*P*-value	<.001	<.001	<.001
	Men			
	β coefficient (95% CI)	−3.89 (−6.92, −0.85)	−3.97 (−7.02, −0.91)	−4.13 (−7.21, −1.06)
	*P*-value	.012	.011	.008
	Women			
	β coefficient (95% CI)	−13.84 (−18.03, −9.65)	−15.33 (−19.64, −11.02)	−15.32 (−19.69, −10.94)
	*P*-value	<.001	<.001	<.001
MPV	Total			
	β coefficient (95% CI)	24.25 (18.03,30.46)	23.31 (17.11,29.51)	21.85 (15.66,28.04)
	*P*-value	<.001	<.001	<.001
	Men			
	β coefficient (95% CI)	26.49 (18.17,34.82)	26.75 (18.42,35.08)	25.22 (16.89,33.55)
	*P*-value	<.001	<.001	<.001
	Women			
	β coefficient (95% CI)	20.38 (11.22,29.55)	19.93 (10.79,29.08)	18.50 (9.37,27.64)
	*P*-value	<.001	<.001	<.001

CRP: C-reactive protein; WBC count: white blood cell count; MPV: mean platelet volume.

Model 1 = unadjusted.

Model 2 = adjusted for age, gender, body mass index, and race/ethnicity.

Model 3 = Model 2 + adjusted for history of angina, coronary heart disease, diabetes mellitus, hypertension, and smoking history.

### Stratification of serum inflammatory biomarkers by quartiles and their associations with s-Klotho levels

**Figure 3.**
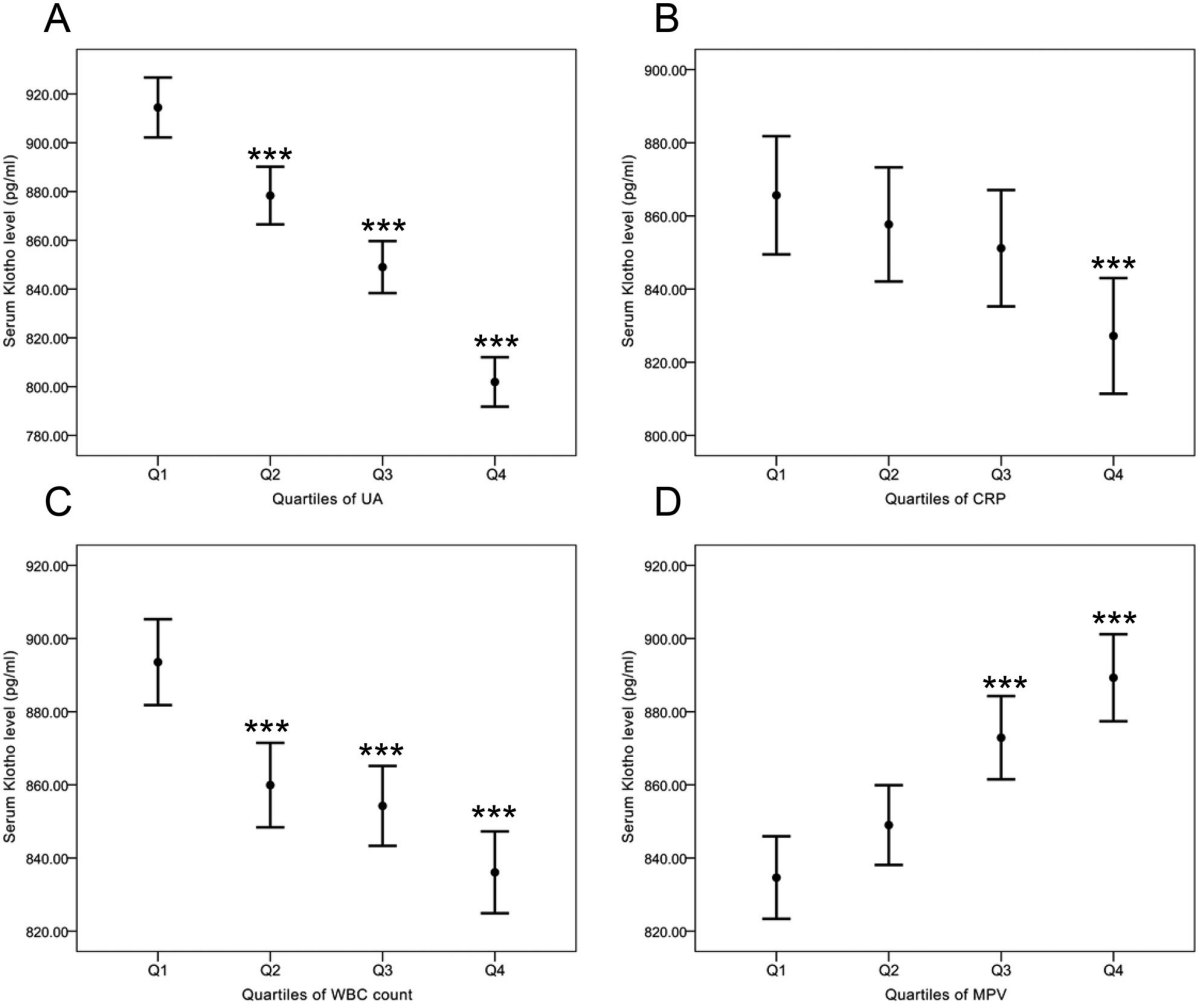
Serum klotho levels in relation to quartiles of inflammation markers. Multivariate linear regression model was used to analyse the associations between the quartiles (Qs) of inflammatory markers (A) UA, (B) CRP, (C) WBC count, (D) MPV and serum klotho level. Serum klotho data are shown as mean + SD. *p<.05, **p<.01, ***p<.001 relative to Quartile 1 of each predictor variable. UA: uric acid; CRP: C-reactive protein; WBC count: white blood cell count; MPV: mean platelet volume

To determine whether the associations between s-Klotho and the four inflammatory biomarkers changed on a gradient, we stratified levels of the four biomarkers into quartiles. In Figure 3(A–C), higher quartiles of UA, CRP, and WBC count correlated with lower levels of s-Klotho, and the lowest level of s-Klotho appeared in Quartile 4 of UA (mean level: 801.91 ± 252.33 mg/dl, not marked in the figure). In contrast,Figure 3(D) reveals that higher quartiles of MPV were correlated with higher levels of s-Klotho. All four biomarkers manifested stronger relationships with s-Klotho levels in the higher quartiles (Table 3). UA especially revealed a strong correlation with s-Klotho levels as the highest absolute values of the β coefficient appeared in Q2 vs. Q1, Q3 vs. Q1, and Q4 vs. Q1 compared with the other biomarkers (Q2 vs. Q1, β coefficient= −35.79, 95% CI= −52.35, −19.22, *p*<.001; Q3 vs. Q1, β coefficient= −65.72, 95% CI= −82.32, −49.13, *p*<.001; Q4 vs. Q1, β coefficient= −107.13, 95% CI= −123.78, −90.48, *p*<.001).

**Table 3.** Multivariate linear regression model for the associations between the quartiles (Qs) of inflammatory markers and serum klotho level.

		Model 1	Model 2	Model 3
Uric acid				
Q2 vs Q1	β coefficient (95% CI)	−35.79 (−52.35, −19.22)	−30.62 (−47.56, −13.68)	−29.22 (−46.12, −12.32)
	*P*-value	<.001	<.001	<.001
Q3 vs Q1	β coefficient (95% CI)	−65.72 (−82.32, −49.13)	−58.47 (−76.29, −40.64)	−55.30 (−73.10, −37.51)
	*P*-value	<.001	<.001	<.001
Q4 vs Q1	β coefficient (95% CI)	−107.13 (−123.78, −90.48)	−99.88 (−118.63, −81.14)	−96.15 (−114.87, −77.43)
	*P*-value	<.001	<.001	<.001
CRP				
Q2 vs Q1	β coefficient (95% CI)	−7.74 (−30.79,15.32)	−7.61 (−30.86,15.63)	−7.52 (−30.70,15.67)
	*P*-value	.511	.521	.525
Q3 vs Q1	β coefficient (95% CI)	−17.81 (−40.74,5.12)	−19.05 (−42.65,4.54)	−19.24 (−42.85,4.36)
	*P*-value	.128	.113	.110
Q4 vs Q1	β coefficient (95% CI)	−39.69 (−62.79, −16.60)	−49.53 (−74.53, −24.53)	−50.30 (−75.39, −25.22)
	*P*-value	.001	<.001	<.001
WBC count				
Q2 vs Q1	β coefficient (95% CI)	−34.02 (−50.64, −17.40)	−31.70 (−48.30, −15.11)	−33.14 (−49.70, −16.59)
	*P*-value	<.001	<.001	<.001
Q3 vs Q1	β coefficient (95% CI)	−38.51 (−55.04, −21.99)	−35.75 (−52.36, −19.13	−37.71 (−54.34, −21.08)
	*P*-value	<.001	<.001	<.001
Q4 vs Q1	β coefficient (95% CI)	−56.39 (−73.16, −39.63)	−57.85 (−74.88, −40.81)	−58.43 (−75.65, −41.21)
	*P*-value	<.001	<.001	<.001
MPV				
Q2 vs Q1	β coefficient (95% CI)	11.64 (−5.04,28.32)	8.35 (−8.25,24.94)	7.56 (−8.97,24.12)
	*P*-value	.171	.324	.370
Q3 vs Q1	β coefficient (95% CI)	33.79 (17.01,50.57)	31.02 (14.31,47.72)	28.90 (12.24,45.56)
	*P*-value	<.001	<.001	.001
Q4 vs Q1	β coefficient (95% CI)	52.94 (36.09,69.79)	50.19 (33.39,66.99)	46.69 (29.93,63.47)
	*P*-value	<.001	<.001	<.001

Q1: Quartile 1; Q2: Quartile 2; Q3: Quartile 3; Q4: Quartile 4; CRP: C-reactive protein; WBC count: white blood cell count; MPV: mean platelet volume.

Model 1 = unadjusted.

Model 2 = adjusted for age, gender, body mass index, and race/ethnicity.

Model 3 = Model 2 + adjusted for history of angina, coronary heart disease, diabetes mellitus, hypertension, and smoking history.

## Discussion

### Highlights of our study

Our study indicates that several established and widely used inflammatory biomarkers are associated with s-Klotho levels. We demonstrated that s-Klotho functions as an inverse indicator of inflammation because of its inverse association with UA, CRP, and WBC count, and its direct association with MPV. Among these, UA was most strongly correlated with s-Klotho. These findings suggest that s-Klotho may serve as a predictive biomarker for inflammation.

### Previous studies that discussed s-Klotho and inflammatory cytokines

Few studies have mentioned the relationship between s-Klotho and inflammatory cytokines. A case–control study with a designated experimental group of 110 patients with histories of cardiovascular disease (CVD) showed that s-Klotho levels were significantly lower in the CVD group and inversely correlated with CRP and TNFα/IL10 [16]. Another study found that s-Klotho was inversely correlated with CRP levels in older populations (≥65 years), but the association was weak (R: −0.245; *p*=.022) [17]. However, these studies were performed in specific groups (either based on disease history or age) rather than in the general population, and the number of biomarkers were limited. Our study provided observations in representative US community-dwelling populations and displayed comparisons between biomarkers and gender differences.

### s-Klotho and UA

Hyperuricaemia has long been associated with gout [18], but recent studies have discovered the importance of asymptomatic hyperuricaemia in kidney disease [19] and CVD [20] through activation of inflammation. The strong relationship between s-Klothoand UA implies that s-Klotho may be indicative of the diseases involving UA, for example in kidney diseases, because both have been described as predictive markers of renal function. Elevated serum UA levels results from decreased renal excretion of uric acid and therefore acts as an independent risk factor for renal function decline [21]. s-Klotho levels are significantly altered in the early stages of chronic kidney disease (CKD), suggesting its utility as an index for early detection of CKD [22]. As for CVD, elevated UA levels are considered risk factors for cardiovascular conditions, including coronary artery disease [23], metabolic syndrome [24], and hypertension [25]. Higher levels of s-Klotho are linked to reduced CVD risk in community-dwelling adults [26] and lower cardio-metabolic risk scores [27]. Investigating the mechanisms underlying inflammation indicated by s-Klotho and UA can infer the strong relationship between them. UA induces TNF-α expression *via* the ROS–MAPK–NF-κB signalling pathway in rat vascular smooth muscle cells, which contributes to the development of CVD [28]. In contrast, s-Klotho inhibits TNF-α–NF-κB activation in endothelial cells, ensuring vascular integrity and cardiovascular health [29]. The mediation of the same pathway by both biomarkers explains how they both regulate inflammation. Moreover, women manifested stronger correlations between UA and s-Klotho than men, illustrating that a unit elevation of UA levels causes a higher increase in s-Klotho levels and inflammation in women. Women have a lower risk of hyperuricaemia compared with men of the same age, owing to the protective effect of oestrogen in UA metabolism [30]. Nevertheless, our results indicate that once women suffer from hyperuricaemia, they are more susceptible to inflammation. Accordingly, the occurrence of hyperuricaemia and low s-Klotho levels in women should be noted in clinical practice as it may indicate a hidden crisis.

### s-Klotho and CRP

The inverse relationship between CRP and s-Klotho has been described in CVD patients in a study performed in Santa Cruz de Tenerife, Spain [16]. However, after matching for age, a decisive factor in s-Klotho levels [31], the Pearson's R no longer showed statistical significance (R = −0.235; *p* = .13). In our study, the inverse association remained significant in all models, including both the unadjusted and fully adjusted ones. This indicated that even after taking the following factors into consideration—age [31], history of CVD(26), and smoking [32]—which all influence and are associated with s-Klotho, the association was still significantly strong. Additionally, the subjects in our study were community-dwelling adults who reflected a relatively healthy population. The above conditions suggest that s-Klotho serves as an independent inflammatory biomarker in the general population.

### s-Klotho and WBC count and MPV

Elevated WBC count, or leukocytosis, is a clinical sign often linked with infection or inflammation [33]. MPV is inversely proportional to platelet count, and increases or decreases of MPV represent various inflammatory processes and diseases [34]. Neither WBC count nor MPV have been mentioned in past studies regarding s-Klotho, but the significant associations determined in our study indicate that decreased s-Klotho levels may be correlated to inflammation, especially in clinical settings when WBC count is elevated and MPV is decreased. Decreased MPV is especially noticeable in inflammatory bowel disease [35], rheumatoid diseases [36], and malignancies [37,38]. s-Klotho may be a useful reference parameter in diagnosis of the abovementioned diseases.

### Role of s-Klotho in hyperglycaemic states

Despite the general negative relationship between s-Klotho levels and inflammatory biomarkers, under certain circumstances, the association may be positive. A recent study measuring serum and urine s-Klotho levels in rats found significantly higher serum levels of s-Klotho in diabetic subjects [39]. Initially, this appeared to conflict with our findings, but closer inspection revealed a possible mechanism explained by the increased Klotho-shedding enzymes, a disintegrin and metalloprotease (ADAM)10 and ADAM17, in diabetic models. Typiak M *et al.* reported that under hyperglycaemic conditions, the shedding of Klotho is expedited, causing a decrease in membrane-bound Klotho and an increase in s-Klotho. They also demonstrated the addition of Klotho to cell medium improved kidney function *via* effects such as increased glycolytic capacity and lowered albumin permeability. Collectively, their findings supported the idea that Klotho ameliorates inflammation, although how s-Klotho predicts inflammatory status in diabetic nephropathy differed from our results. In view of this, the interpretation of serum s-Klotho levels in specific diseases requires further investigation.

### Molecular mechanisms of s-Klotho in modulation of inflammation

Levels of biomarkers discussed in the present study were altered significantly during the inflammatory process; however, each was regulated in by a unique mechanism. Elevation in circulating WBCs indicates the initiation of host defences against various kinds of injuries, and the five types of WBCs, neutrophils, lymphocytes, eosinophils, basophils, and monocytes, share general anti-inflammatory properties, although each mediates specific immune responses [40]. Increased UA levels are thought to be independent risk factors for CVD because UA activates vascular smooth muscle cell proliferation, triggers the release of inflammatory cytokines (TNF-α, IL-1, and IFN-γ), and contributes to reactive oxygen species(ROS)-mediated damage [41]. CRP is primarily synthesized in hepatocytes, and its circulating concentration increases during the acute inflammatory phase as it responds to IL-6 secreted by macrophages and T cells and further activates the complement system. In short, CRP itself is a downstream mediator within inflammatory signalling pathways and is therefore detectable in various inflammatory conditions [42].

Numerous studies that have investigated the functions of Klotho have demonstrated its distinctive role in vascular homeostasis. In CVD, Klotho suppresses IL-6 production in endothelial cells [43], inhibiting TRPC6 channels in cardiomyocytes [44], and inactivates ROS and NF-κB-mediated inflammation [45]. In the kidneys, Klotho reduces podocyte injuries through inhibition of insulin-like growth factor 1, protein kinase-1/2, and p38 mitogen-activated protein kinase [46]. It also increases fibroblast growth factor receptor levels and glycolytic capacity and decreases glomerular albumin permeability in hyperglycemia [39]. Accordingly, the role of s-Klotho as an inverse indicator of inflammation may be more prominent in cardiovascular and kidney-related diseases. Future studies are required to enhance our understanding of s-Klotho levels in different diseases.

### Comparison of four inflammatory markers

Comparing the four biomarkers led to the noteworthy finding that UA levels had the strongest relationship with s-Klotho levels, which may be attributable to their shared site of production—the kidney. Serum UA level is determined mainly by reabsorption and excretion in the kidney [47], and s-Klotho is produced by shedding of the extracellular domain of membrane-bound Klotho in renal tubules [48]. Nevertheless, because the four inflammatory biomarkers all exhibited significant associations in unadjusted and fully adjusted models, we believe s-Klotho levels are capable of representing a general inflammation status rather than kidney-specific disorders.

The pros and cons of incorporating s-Klotho testing into routine clinical practice are worth discussing. Compared with UA, CRP, WBC count, and MPV, which are already commonly used laboratory markers, the importance of Klotho has only been determined in the past decade. While the other four markers are altered in most inflammatory conditions, s-Klotho stands out because it is regulated in particular organs, such as the kidneys, and in cardiovascular-involved diseases, which permits it to provide distinct diagnostic values. However, Klotho is more costly to determine than established biomarkers, which may limit its application in general practice at the present time.

### Limitations

The findings of this study must be viewed considering some limitations. First, our study revealed a positive relationship between s-Klotho and MPV, illustrating that decreased MPV is linked to inflammation. However, studies have shown that either increases or decreases in MPV can indicate various inflammatory statuses and diseases [34]. Interpretation of MPV should be combined with other laboratory results, such as complete blood count tests, and take factors affecting MPV into account, such as smoking [49] or menstruation [50]. Prediction of a disease by MPV alone or recognising that only decreases in MPV correlate with inflammation may result in biased conclusions. Furthermore, NHANES is a cross-sectional survey; hence, it is not possible to establish causal relationships. We are unable to determine the direction of cause and effect between s-Klotho and the four inflammatory biomarkers. Moreover, several essential modulating factors that affect s-Klotho levels, such as renal function decline (the estimated glomerular filtration rate) and the usage of anti-hypertensive agents (especially those targeting the renin–angiotensinsystem) are not available in the NHANES database. Hence, we did not include these potential confounders in the fully adjusted models. Further studies are warranted to verify the precise associations between s-Klotho and UA, CRP, WBC count, and MPV to allow more extensive discussions about this issue.

## Conclusion

s-Klotho is significantly associated with well-recognized inflammatory biomarkers. Negative associations were demonstrated with UA, CRP, and WBC count, while a positive association was demonstrated with MPV. UA had the strongest relationship with s-Klotho, suggesting that s-Klotho could serve as an alternative inverse indicator for evaluation of inflammatory states. Further studies are required to assess the applicability of s-Klotho in clinical practice.

## Data Availability

The datasets generated and/or analysed during the current study are publicly available from the NHANES website. (https://wwwn.cdc.gov/nchs/nhanes/nhanes3/default.aspx).
